# Supplementary material for: The mediating roles of coping styles and academic burnout in the relationship between stressors and depressive symptoms among Chinese postgraduates
Source: PeerJ. 2023 Sep 18;11:e16064. doi: 10.7717/peerj.16064 (PMC10512960; doi:10.7717/peerj.16064)
Supplement: Supplemental Information 1 — The results adjusted for gender, age (year), registered residence, the only child, average monthly household income (yuan), average monthly living expenses (yuan). _a Unstandardized regression coefficients, and _b Standardized regression coefficients. [file peerj-11-16064-s001.docx]

**Supplementary Table S1:**

The results of the path coefficients for the path analysis.

| **Model pathways** | **Estimate^a^** | **S.E.** | **C.R.** | ***P*** | **Estimate^b^** |
| --- | --- | --- | --- | --- | --- |
| Stressors →Positive coping style | -0.01 | 0.00 | -3.49 | <0.001 | -0.21 |
| Stressors →Negative coping style | 0.01 | 0.00 | 4.33 | <0.001 | 0.26 |
| Stressors →Academic burnout | 0.26 | 0.03 | 6.23 | <0.001 | 0.33 |
| Stressors →Depressive symptoms | 0.17 | 0.03 | 6.08 | <0.001 | 0.31 |
| Positive coping style →Academic burnout | -7.11 | 1.31 | -5.41 | <0.001 | -0.27 |
| Negative coping style →Academic burnout | 5.64 | 1.02 | 5.54 | <0.001 | 0.28 |
| Positive coping style →Depressive symptoms | -4.60 | 1.10 | -4.17 | <0.001 | -0.20 |
| Negative coping style →Depressive symptoms | 2.29 | 0.86 | 2.67 | 0.008 | 0.13 |
| Academic burnout →Depressive symptoms | 0.29 | 0.05 | 5.84 | <0.001 | 0.32 |

Notes.

The results adjusted for gender, age (year), registered residence, the only child, average monthly household income (yuan), average monthly living expenses (yuan).

^a^ Unstandardized regression coefficients, and ^b^ Standardized regression coefficients.
