# Supplementary material for: The mediating roles of coping styles and academic burnout in the relationship between stressors and depressive symptoms among Chinese postgraduates
Source: PeerJ. 2023 Sep 18;11:e16064. doi: 10.7717/peerj.16064 (PMC10512960; doi:10.7717/peerj.16064)
Supplement: Supplemental Information 3 [file peerj-11-16064-s003.docx]

**The codebook that converts numbers to their respective factors is as follows:**

| Variables | Code | Code | Code |
| --- | --- | --- | --- |
| Gender | 1= “Male” | 2= “Female” |  |
| Age (year) (continuous) |  |  |  |
| The Only child | 1= “Yes” | 2= “No” |  |
| Registered residence | 1= “Urban” | 2= “Rural” |  |
| Average monthly household income (yuan) | 1= “≤3000” | 2= “3001-5000” | 3= “≥5001” |
| Average monthly living expenses (yuan) | 1= “≤1500” | 2= “1501-2000” | 3= “≥2001” |
| Stress (continuous) |  |  |  |
| Positive coping style (continuous) |  |  |  |
| Negative coping style (continuous) |  |  |  |
| Academic burnout (continuous) |  |  |  |
| Depression symptoms (continuous) |  |  |  |
