# Supplementary material for: The mediating roles of coping styles and academic burnout in the relationship between stressors and depressive symptoms among Chinese postgraduates
Source: PeerJ. 2023 Sep 18;11:e16064. doi: 10.7717/peerj.16064 (PMC10512960; doi:10.7717/peerj.16064)
Supplement: Supplemental Information 4 [file peerj-11-16064-s004.doc]

**研究生健康信息采集表**

**个人基本情况（A）**

**A1.性别：①男 ②女**

**A2.出生日期（阳历）： 年 月 日**

**A3.您是否为独生子女：①是 ②否**

**A4.家庭所在地：①城市 ②城镇 ③农村**

**A5.最近一年，您平均每月的生活开销**

**①<500元 ②501-1000元 ③1001-1500元**

**④1501-2000元 ⑤>2001元**

**A6.最近一年，您家庭的人均月收入：**

**①1000及以下 ②1001-3000 ③3001-5000 ④5001及以上**

**测量（B）**

**请您结合最近一周的实际情况，在最符合您的选项中打“√”。**

**B1.我觉得闷闷不乐，情绪低沉**

**①没有或很少 ②有时 ③大部分时间 ④绝大部分时间有**

**B2.我觉得一天之中早晨心情最好**

**①没有或很少 ②有时 ③大部分时间 ④绝大部分时间有**

**B3.我一阵阵哭出来或觉得想哭**

**①没有或很少 ②有时 ③大部分时间 ④绝大部分时间有**

**B4.我晚上睡眠不好**

**①没有或很少 ②有时 ③大部分时间 ④绝大部分时间有**

**B5.我吃得跟平常一样多**

**①没有或很少 ②有时 ③大部分时间 ④绝大部分时间有**

**B6.我与异性亲密接触时和以往一样感到愉快**

**①没有或很少 ②有时 ③大部分时间 ④绝大部分时间有**

**B7.我发觉我的体重在下降**

**①没有或很少 ②有时 ③大部分时间 ④绝大部分时间有**

**B8.我有便秘的苦恼**

**①没有或很少 ②有时 ③大部分时间 ④绝大部分时间有**

**B9.我心跳比平时快**

**①没有或很少 ②有时 ③大部分时间 ④绝大部分时间有**

**B10.我无缘无故感到疲乏**

**①没有或很少 ②有时 ③大部分时间 ④绝大部分时间有**

**B11.我的头脑跟平常一样清楚**

**①没有或很少 ②有时 ③大部分时间 ④绝大部分时间有**

**B12.我觉得做以前经常做的事并没有困难**

**①没有或很少 ②有时 ③大部分时间 ④绝大部分时间有**

**B13.我坐立不安，难以保持平静**

**①没有或很少 ②有时 ③大部分时间 ④绝大部分时间有**

**B14.我对将来抱有希望**

**①没有或很少 ②有时 ③大部分时间 ④绝大部分时间有**

**B15.我比平常容易激动**

**①没有或很少 ②有时 ③大部分时间 ④绝大部分时间有**

**B16.我觉得做出决定是容易的**

**①没有或很少 ②有时 ③大部分时间 ④绝大部分时间有**

**B17.我觉得自己是个有用的人，有人需要我**

**①没有或很少 ②有时 ③大部分时间 ④绝大部分时间有**

**B18.我的生活过得很有意思**

**①没有或很少 ②有时 ③大部分时间 ④绝大部分时间有**

**B19.我认为如果我死了别人会生活得好些**

**①没有或很少 ②有时 ③大部分时间 ④绝大部分时间有**

**B20.平常感兴趣的事我仍然照样感兴趣**

**①没有或很少 ②有时 ③大部分时间 ④绝大部分时间有**

**测量（C）**

**近半年内，请根据下列事件给您造成的苦恼程度在第②至第⑥项中打“√”；如果该事件未发生，则在“未发生”一项打“√”。**

**C1.学习内容或专业单调枯燥**

**①未发生 ②发生过但没影响 ③轻度影响**

**④中度影响 ⑤重度影响 ⑥极重影响**

**C2.不满学校的培养模式和课程设置**

**①未发生 ②发生过但没影响 ③轻度影响**

**④中度影响 ⑤重度影响 ⑥极重影响**

**C3.课题研究进展不顺利**

**①未发生 ②发生过但没影响 ③轻度影响**

**④中度影响 ⑤重度影响 ⑥极重影响**

**C4.他人对自己期望比以前高**

**①未发生 ②发生过但没影响 ③轻度影响**

**④中度影响 ⑤重度影响 ⑥极重影响**

**C5.日常支出导致经济压力**

**①未发生 ②发生过但没影响 ③轻度影响**

**④中度影响 ⑤重度影响 ⑥极重影响**

**C6.社交活动类型或数量有较大的变化**

**①未发生 ②发生过但没影响 ③轻度影响**

**④中度影响 ⑤重度影响 ⑥极重影响**

**C7.被人误会或错怪**

**①未发生 ②发生过但没影响 ③轻度影响**

**④中度影响 ⑤重度影响 ⑥极重影响**

**C8.学费压力**

**①未发生 ②发生过但没影响 ③轻度影响**

**④中度影响 ⑤重度影响 ⑥极重影响**

**C9.较大地改变个人生活习惯（如饮食、睡眠）**

**①未发生 ②发生过但没影响 ③轻度影响**

**④中度影响 ⑤重度影响 ⑥极重影响**

**C10.专业不好，就业前景不利**

**①未发生 ②发生过但没影响 ③轻度影响**

**④中度影响 ⑤重度影响 ⑥极重影响**

**C11.家庭经济状况不佳**

**①未发生 ②发生过但没影响 ③轻度影响**

**④中度影响 ⑤重度影响 ⑥极重影响**

**C12.兼职或助管工作不理想**

**①未发生 ②发生过但没影响 ③轻度影响**

**④中度影响 ⑤重度影响 ⑥极重影响**

**C13.就业压力大**

**①未发生 ②发生过但没影响 ③轻度影响**

**④中度影响 ⑤重度影响 ⑥极重影响**

**C14.奖学金或其它奖项落空**

**①未发生 ②发生过但没影响 ③轻度影响**

**④中度影响 ⑤重度影响 ⑥极重影响**

**C15.不得不参加某些社交活动**

**①未发生 ②发生过但没影响 ③轻度影响**

**④中度影响 ⑤重度影响 ⑥极重影响**

**C16.恋爱或失恋**

**①未发生 ②发生过但没影响 ③轻度影响**

**④中度影响 ⑤重度影响 ⑥极重影响**

**C17.个人借贷**

**①未发生 ②发生过但没影响 ③轻度影响**

**④中度影响 ⑤重度影响 ⑥极重影响**

**C18.学习方式的较大转变**

**①未发生 ②发生过但没影响 ③轻度影响**

**④中度影响 ⑤重度影响 ⑥极重影响**

**C19.与恋人发生矛盾**

**①未发生 ②发生过但没影响 ③轻度影响**

**④中度影响 ⑤重度影响 ⑥极重影响**

**C20.某些课程单调乏味，但不得不上**

**①未发生 ②发生过但没影响 ③轻度影响**

**④中度影响 ⑤重度影响 ⑥极重影响**

**C21.较大地改变个人的价值观或自我评价**

**①未发生 ②发生过但没影响 ③轻度影响**

**④中度影响 ⑤重度影响 ⑥极重影响**

**C22.社会竞争日趋激烈，担心就业**

**①未发生 ②发生过但没影响 ③轻度影响**

**④中度影响 ⑤重度影响 ⑥极重影响**

**C23.自己生病或受伤**

**①未发生 ②发生过但没影响 ③轻度影响**

**④中度影响 ⑤重度影响 ⑥极重影响**

**C24.有关性的困扰**

**①未发生 ②发生过但没影响 ③轻度影响**

**④中度影响 ⑤重度影响 ⑥极重影响**

**C25.担心自己能力不强从而影响就业**

**①未发生 ②发生过但没影响 ③轻度影响**

**④中度影响 ⑤重度影响 ⑥极重影响**

**C26.与他人产生矛盾**

**①未发生 ②发生过但没影响 ③轻度影响**

**④中度影响 ⑤重度影响 ⑥极重影响**

**C27.对发表论文的担心**

**①未发生 ②发生过但没影响 ③轻度影响**

**④中度影响 ⑤重度影响 ⑥极重影响**

**C28.亲人或朋友遇到困难**

**①未发生 ②发生过但没影响 ③轻度影响**

**④中度影响 ⑤重度影响 ⑥极重影响**

**C29.关于找男（女）朋友的困惑**

**①未发生 ②发生过但没影响 ③轻度影响**

**④中度影响 ⑤重度影响 ⑥极重影响**

**C30.相比之下，科研条件不好**

**①未发生 ②发生过但没影响 ③轻度影响**

**④中度影响 ⑤重度影响 ⑥极重影响**

**C31.导师缺少课题或指导不利**

**①未发生 ②发生过但没影响 ③轻度影响**

**④中度影响 ⑤重度影响 ⑥极重影响**

**测量（D）**

**D1.我有自己的学习方法和计划，并能付诸实践**

**①完全不符合 ②不符合 ③不确定 ④符合 ⑤完全符合**

**D2.我觉得所学的知识毫无用处**

**①完全不符合 ②不符合 ③不确定 ④符合 ⑤完全符合**

**D3.专业知识的掌握对我来说很容易**

**①完全不符合 ②不符合 ③不确定 ④符合 ⑤完全符合**

**D4.清早起來，想到要面对一天的学习，便感到很疲倦**

**①完全不符合 ②不符合 ③不确定 ④符合 ⑤完全符合**

**D5.我很难对学习保持长久的热情**

**①完全不符合 ②不符合 ③不确定 ④符合 ⑤完全符合**

**D6.我通常能静下心来认真学习**

**①完全不符合 ②不符合 ③不确定 ④符合 ⑤完全符合**

**D7.整天学下来，我感到筋疲力尽**

**①完全不符合 ②不符合 ③不确定 ④符合 ⑤完全符合**

**D8.到目前为止，研究生学习使我的能力得到充分的展示**

**①完全不符合 ②不符合 ③不确定 ④符合 ⑤完全符合**

**D9.我对学习感到厌倦**

**①完全不符合 ②不符合 ③不确定 ④符合 ⑤完全符合**

**D10.我课后很少学习**

**①完全不符合 ②不符合 ③不确定 ④符合 ⑤完全符合**

**D11.我能胜任硕士/博士阶段的学术研究**

**①完全不符合 ②不符合 ③不确定 ④符合 ⑤完全符合**

**D12.我学习时经常打磕睡**

**①完全不符合 ②不符合 ③不确定 ④符合 ⑤完全符合**

**D13.我对自己的专业很感兴趣**

**①完全不符合 ②不符合 ③不确定 ④符合 ⑤完全符合**

**D14.在学习上我觉得我耐性还不够**

**①完全不符合 ②不符合 ③不确定 ④符合 ⑤完全符合**

**D15.对我来说，拿到硕士/博士学位很容易**

**①完全不符合 ②不符合 ③不确定 ④符合 ⑤完全符合**

**D16.只有导师督促时，我才会学习**

**①完全不符合 ②不符合 ③不确定 ④符合 ⑤完全符合**

**D17.我想做些研究，但感到研究很枯燥**

**①完全不符合 ②不符合 ③不确定 ④符合 ⑤完全符合**

**D18.学习时我精力充沛**

**①完全不符合 ②不符合 ③不确定 ④符合 ⑤完全符合**

**D19.我很少计划、安排自己的学习时间**

**①完全不符合 ②不符合 ③不确定 ④符合 ⑤完全符合**

**D20.课程/科研任务总是让我厌烦**

**①完全不符合 ②不符合 ③不确定 ④符合 ⑤完全符合**

**测量(E)**

**下列题目中是生活中遇到困难时可能采取的态度和做法，请仔细阅读每一项，然后在选择最符合自身情况的选项作答。**

**E1.通过工作学习或一些其他活动解脱**

**①不采取 ②偶尔采取 ③有时采取 ④经常采取**

**E2.与人交谈,倾诉内心烦恼**

**①不采取 ②偶尔采取 ③有时采取 ④经常采取**

**E3.尽量看到事物好的一面**

**①不采取 ②偶尔采取 ③有时采取 ④经常采取**

**E4.改变自己的想法，重新发现生活中什么重要**

**①不采取 ②偶尔采取 ③有时采取 ④经常采取**

**E5.不把问题看得太严重**

**①不采取 ②偶尔采取 ③有时采取 ④经常采取**

**E6.坚持自己的立场,为自己想得到的斗争**

**①不采取 ②偶尔采取 ③有时采取 ④经常采取**

**E7.找出几种不同的解决问题的方法**

**①不采取 ②偶尔采取 ③有时采取 ④经常采取**

**E8.向家人、亲戚朋友或同学寻求建议**

**①不采取 ②偶尔采取 ③有时采取 ④经常采取**

**E9.改变原来的一些做法或改正自己的一些问题**

**①不采取 ②偶尔采取 ③有时采取 ④经常采取**

**E10.借鉴他人处理类似困难情境的办法**

**①不采取 ②偶尔采取 ③有时采取 ④经常采取**

**E11.寻找业余爱好，积极参加文体活动**

**①不采取 ②偶尔采取 ③有时采取 ④经常采取**

**E12.尽量克制自己的失望、悔恨、悲伤或愤怒**

**①不采取 ②偶尔采取 ③有时采取 ④经常采取**

**E13.试图休息或休假,暂时把问题（烦恼）抛开**

**①不采取 ②偶尔采取 ③有时采取 ④经常采取**

**E14.通过抽烟、喝酒、服药、吃东西等来解除烦恼**

**①不采取 ②偶尔采取 ③有时采取 ④经常采取**

**E15.认为时间会改变现状,唯一要做的便是等待**

**①不采取 ②偶尔采取 ③有时采取 ④经常采取**

**E16.试图忘记整个事情**

**①不采取 ②偶尔采取 ③有时采取 ④经常采取**

**E17.依靠别人解决问题**

**①不采取 ②偶尔采取 ③有时采取 ④经常采取**

**E18.接受现实,因为没有什么别的办法**

**①不采取 ②偶尔采取 ③有时采取 ④经常采取**

**E19.幻想可能会发生某种奇迹改变现状**

**①不采取 ②偶尔采取 ③有时采取 ④经常采取**

**E20.自己安慰自己**

**①不采取 ②偶尔采取 ③有时采取 ④经常采取**
