# Supplementary material for: The mediating roles of coping styles and academic burnout in the relationship between stressors and depressive symptoms among Chinese postgraduates
Source: PeerJ. 2023 Sep 18;11:e16064. doi: 10.7717/peerj.16064 (PMC10512960; doi:10.7717/peerj.16064)
Supplement: Supplemental Information 5 [file peerj-11-16064-s005.doc]

**Postgraduate health information questionnaire**

**Personal basic information (A)**

A1. Gender: ① Male ② Female

A2. Date of birth (Gregorian calendar): MM DD YY

A3. Are you an only child? ① Yes ② No.

A4. Home location: ① City ② Town ③ Countryside

A5. In the last year, your average monthly living expenses?

①<500 yuan ②501-1000 yuan ③1001-1500 yuan ④1501-2000 yuan ⑤>2001 yuan

A6. In the last year, the per capita monthly income of your family:

①1000 and below ②1001-3000 ③3001-5000 ④5001 and above

**Measurement (B)**

**Please tick “√” in the most suitable option according to the actual situation in the last week.**

B1. I feel down-hearted and blue

① A little of the time ② Some of the time

③ Good part of the time ④ Most of the time

B2. Morning is when l fell the best

① A little of the time ② Some of the time

③ Good part of the time ④ Most of the time

B3. I have crying spells or feel like it

① A little of the time ② Some of the time

③ Good part of the time ④ Most of the time

B4. I have trouble sleeping at night

① A little of the time ② Some of the time

③ Good part of the time ④ Most of the time

B5. I eat as much as I used to

① A little of the time ② Some of the time

③ Good part of the time ④ Most of the time

B6. I still enjoy sex

① A little of the time ② Some of the time

③ Good part of the time ④ Most of the time

B7. I notice that I am losing weight

① A little of the time ② Some of the time

③ Good part of the time ④ Most of the time

B8. I have trouble with constipation

① A little of the time ② Some of the time

③ Good part of the time ④ Most of the time

B9. My heart beats faster than usual

① A little of the time ② Some of the time

③ Good part of the time ④ Most of the time

B10. I get tired for no reason

① A little of the time ② Some of the time

③ Good part of the time ④ Most of the time

B11. My mind is as clear as it used to be

① A little of the time ② Some of the time

③ Good part of the time ④ Most of the time

B12. I find it easy to do things I used to

① A little of the time ② Some of the time

③ Good part of the time ④ Most of the time

B13. I am restless and can’t keep still

① A little of the time ② Some of the time

③ Good part of the time ④ Most of the time

B14. I fell hopeful about the future

① A little of the time ② Some of the time

③ Good part of the time ④ Most of the time

B15. I am more irritable than usual

① A little of the time ② Some of the time

③ Good part of the time ④ Most of the time

B16. I find it easy to make decisions

① A little of the time ② Some of the time

③ Good part of the time ④ Most of the time

B17. I fell that I am useful and needed

① A little of the time ② Some of the time

③ Good part of the time ④ Most of the time

B18. My life is pretty full

① A little of the time ② Some of the time

③ Good part of the time ④ Most of the time

B19. I fell that others would be better off if I were dead

① A little of the time ② Some of the time

③ Good part of the time ④ Most of the time

B20. I still enjoy the things I used to do

① A little of the time ② Some of the time

③ Good part of the time ④ Most of the time

**Measurement (C)**

**In the past six months, please tick “√” in items ② to ⑥ according to the distress caused by the following events; If the event does not occur, tick “√” in the “Not Happened” item.**

C1. Learning content or professional drab

① Not Happened ② It happened, but it didn’t affect ③ Mild effect ④ Moderate effect ⑤ Severe effect ⑥ Extremely severe effect

C2. Dissatisfied with the school’s training model and curriculum

① Not Happened ② It happened, but it didn’t affect ③ Mild effect ④ Moderate effect ⑤ Severe effect ⑥ Extremely severe effect

C3. Project research progress is not smooth

① Not Happened ② It happened, but it didn’t affect ③ Mild effect ④ Moderate effect ⑤ Severe effect ⑥ Extremely severe effect

C4. Others expect more than before

① Not Happened ② It happened, but it didn’t affect ③ Mild effect ④ Moderate effect ⑤ Severe effect ⑥ Extremely severe effect

C5. Daily expenses lead to economic pressure

① Not Happened ② It happened, but it didn’t affect ③ Mild effect ④ Moderate effect ⑤ Severe effect ⑥ Extremely severe effect

C6. There are significant changes in the type or number of social activities

① Not Happened ② It happened, but it didn’t affect ③ Mild effect ④ Moderate effect ⑤ Severe effect ⑥ Extremely severe effect

C7. Be misunderstood or wrong

① Not Happened ② It happened, but it didn’t affect ③ Mild effect ④ Moderate effect ⑤ Severe effect ⑥ Extremely severe effect

C8. Tuition pressure

① Not Happened ② It happened, but it didn’t affect ③ Mild effect ④ Moderate effect ⑤ Severe effect ⑥ Extremely severe effect

C9. Major changes in personal habits (such as diet, sleep)

① Not Happened ② It happened, but it didn’t affect ③ Mild effect ④ Moderate effect ⑤ Severe effect ⑥ Extremely severe effect

C10. Poor professional, unfavorable employment prospects

① Not Happened ② It happened, but it didn’t affect ③ Mild effect ④ Moderate effect ⑤ Severe effect ⑥ Extremely severe effect

C11. Poor family finances

① Not Happened ② It happened, but it didn’t affect ③ Mild effect ④ Moderate effect ⑤ Severe effect ⑥ Extremely severe effect

C12. Part-time or assistant management work is not ideal

① Not Happened ② It happened, but it didn’t affect ③ Mild effect ④ Moderate effect ⑤ Severe effect ⑥ Extremely severe effect

C13. High employment pressure

① Not Happened ② It happened, but it didn’t affect ③ Mild effect ④ Moderate effect ⑤ Severe effect ⑥ Extremely severe effect

C14. Failure of Scholarship or Other Awards

① Not Happened ② It happened, but it didn’t affect ③ Mild effect ④ Moderate effect ⑤ Severe effect ⑥ Extremely severe effect

C15. Forced to engage in certain social activities

① Not Happened ② It happened, but it didn’t affect ③ Mild effect ④ Moderate effect ⑤ Severe effect ⑥ Extremely severe effect

C16. Love or brokenhearted

① Not Happened ② It happened, but it didn’t affect ③ Mild effect ④ Moderate effect ⑤ Severe effect ⑥ Extremely severe effect

C17. Individual borrowing

① Not Happened ② It happened, but it didn’t affect ③ Mild effect ④ Moderate effect ⑤ Severe effect ⑥ Extremely severe effect

C18. Major changes in learning styles

① Not Happened ② It happened, but it didn’t affect ③ Mild effect ④ Moderate effect ⑤ Severe effect ⑥ Extremely severe effect

C19. Conflicts with lovers

① Not Happened ② It happened, but it didn’t affect ③ Mild effect ④ Moderate effect ⑤ Severe effect ⑥ Extremely severe effect

C20. Some courses are tedious but have to be taken

① Not Happened ② It happened, but it didn’t affect ③ Mild effect ④ Moderate effect ⑤ Severe effect ⑥ Extremely severe effect

C21. Major changes in personal values or self-evaluation

① Not Happened ② It happened, but it didn’t affect ③ Mild effect ④ Moderate effect ⑤ Severe effect ⑥ Extremely severe effect

C22. Social competition is increasingly fierce, worried about employment

① Not Happened ② It happened, but it didn’t affect ③ Mild effect ④ Moderate effect ⑤ Severe effect ⑥ Extremely severe effect

C23. Illness or injury of your own

① Not Happened ② It happened, but it didn’t affect ③ Mild effect ④ Moderate effect ⑤ Severe effect ⑥ Extremely severe effect

C24. Sexual harassment

① Not Happened ② It happened, but it didn’t affect ③ Mild effect ④ Moderate effect ⑤ Severe effect ⑥ Extremely severe effect

C25. Worry about your lack of ability that affects your employment

① Not Happened ② It happened, but it didn’t affect ③ Mild effect ④ Moderate effect ⑤ Severe effect ⑥ Extremely severe effect

C26. Conflict with others

① Not Happened ② It happened, but it didn’t affect ③ Mild effect ④ Moderate effect ⑤ Severe effect ⑥ Extremely severe effect

C27. Worries about Publishing a Paper

① Not Happened ② It happened, but it didn’t affect ③ Mild effect ④ Moderate effect ⑤ Severe effect ⑥ Extremely severe effect

C28. Difficulties faced by relatives or friends

① Not Happened ② It happened, but it didn’t affect ③ Mild effect ④ Moderate effect ⑤ Severe effect ⑥ Extremely severe effect

C29. Confusion about finding male (female) friends

① Not Happened ② It happened, but it didn’t affect ③ Mild effect ④ Moderate effect ⑤ Severe effect ⑥ Extremely severe effect

C30. By contrast, scientific research conditions are bad

① Not Happened ② It happened, but it didn’t affect ③ Mild effect ④ Moderate effect ⑤ Severe effect ⑥ Extremely severe effect

C31. Lack of project or unfavorable guidance from mentor

① Not Happened ② It happened, but it didn’t affect ③ Mild effect ④ Moderate effect ⑤ Severe effect ⑥ Extremely severe effect

**Measurement (D)**

D1. I have my own learning methods and plans, and can put them into practice

① Completely inconsistent ② Incompatible ③ Uncertain ④ Consistent

⑤ Completely consistent

D2. I think what I have learned is useless

① Completely inconsistent ② Incompatible ③ Uncertain ④ Consistent

⑤ Completely consistent

D3. Mastering professional knowledge is easy for me

① Completely inconsistent ② Incompatible ③ Uncertain ④ Consistent

⑤ Completely consistent

D4. When I get up early in the morning, I feel very tired at the thought of facing a day of study

① Completely inconsistent ② Incompatible ③ Uncertain ④ Consistent

⑤ Completely consistent

D5. It's hard for me to keep a passion for learning for a long time

① Completely inconsistent ② Incompatible ③ Uncertain ④ Consistent

⑤ Completely consistent

D6.I can usually settle down and study hard

① Completely inconsistent ② Incompatible ③ Uncertain ④ Consistent

⑤ Completely consistent

D7.I feel exhausted after learning all day

① Completely inconsistent ② Incompatible ③ Uncertain ④ Consistent

⑤ Completely consistent

D8. So far, my abilities have been fully demonstrated by the postgraduate study

① Completely inconsistent ② Incompatible ③ Uncertain ④ Consistent

⑤ Completely consistent

D9.I am tired of studying

① Completely inconsistent ② Incompatible ③ Uncertain ④ Consistent

⑤ Completely consistent

D10. I seldom study after class

① Completely inconsistent ② Incompatible ③ Uncertain ④ Consistent

⑤ Completely consistent

D11. I am qualified for academic research at the master's/doctoral level

① Completely inconsistent ② Incompatible ③ Uncertain ④ Consistent

⑤ Completely consistent

D12. I often doze off when I study

① Completely inconsistent ② Incompatible ③ Uncertain ④ Consistent

⑤ Completely consistent

D13. I am very interested in my major

① Completely inconsistent ② Incompatible ③ Uncertain ④ Consistent

⑤ Completely consistent

D14. I don't think I have enough patience in my studies

① Completely inconsistent ② Incompatible ③ Uncertain ④ Consistent

⑤ Completely consistent

D15. It's easy for me to get a master's/doctor's degree

① Completely inconsistent ② Incompatible ③ Uncertain ④ Consistent

⑤ Completely consistent

D16. I will study only when my tutor urges me

① Completely inconsistent ② Incompatible ③ Uncertain ④ Consistent

⑤ Completely consistent

D17. I want to do some research, but find it boring

① Completely inconsistent ② Incompatible ③ Uncertain ④ Consistent

⑤ Completely consistent

D18. I am full of energy when I study

① Completely inconsistent ② Incompatible ③ Uncertain ④ Consistent

⑤ Completely consistent

D19. I seldom plan and arrange my study time

① Completely inconsistent ② Incompatible ③ Uncertain ④ Consistent

⑤ Completely consistent

D20. Course/research assignments always bore me

① Completely inconsistent ② Incompatible ③ Uncertain ④ Consistent

⑤ Completely consistent

**Measurement (E)**

**The following topics are the attitudes and practices that you may adopt when you encounter difficulties in your life. Please read each item carefully, and then choose the option that best suits your situation.**

E1. Release through work study or some other activity

① Don't take ② Occasionally take ③ Sometimes take ④ Often take

E2. Talk to people, pour out inner troubles

① Don't take ② Occasionally take ③ Sometimes take ④ Often take

E3. Try to look on the bright side

① Don't take ② Occasionally take ③ Sometimes take ④ Often take

E4. Change your mind and rediscover what's important in life

① Don't take ② Occasionally take ③ Sometimes take ④ Often take

E5. Don't take the problem too seriously

① Don't take ② Occasionally take ③ Sometimes take ④ Often take

E6. Stand your ground and fight for what you want

① Don't take ② Occasionally take ③ Sometimes take ④ Often take

E7. Find several different solutions to the problem

① Don't take ② Occasionally take ③ Sometimes take ④ Often take

E8. Seek advice from family, relatives, friends, or classmates

① Don't take ② Occasionally take ③ Sometimes take ④ Often take

E9. Change some of the original practices or correct some of their own problems

① Don't take ② Occasionally take ③ Sometimes take ④ Often take

E10. Learn from others' approaches to similar difficult situations

① Don't take ② Occasionally take ③ Sometimes take ④ Often take

E11. Find hobbies, and actively participate in sports activities

① Don't take ② Occasionally take ③ Sometimes take ④ Often take

E12. Try to control your disappointment, regret, sadness, or anger

① Don't take ② Occasionally take ③ Sometimes take ④ Often take

E13. Try to take a rest or vacation to temporarily put the problem (worry) aside

① Don't take ② Occasionally take ③ Sometimes take ④ Often take

E14. Remove troubles by smoking, drinking, taking medicine, and eating

① Don't take ② Occasionally take ③ Sometimes take ④ Often take

E15. Think time will change the status quo, the only thing to do is to wait

① Don't take ② Occasionally take ③ Sometimes take ④ Often take

E16. Try to forget the whole thing

① Don't take ② Occasionally take ③ Sometimes take ④ Often take

E17. Relying on others to solve problems

① Don't take ② Occasionally take ③ Sometimes take ④ Often take

E18. Accept the reality, because there is no other way

① Don't take ② Occasionally take ③ Sometimes take ④ Often take

E19. Imagine that some miracle might happen to change the situation

① Don't take ② Occasionally take ③ Sometimes take ④ Often take

E20. Comfort yourself

① Don't take ② Occasionally take ③ Sometimes take ④ Often take
